# Supplementary material for: 24 hour consultant obstetrician presence on the labour ward and intrapartum outcomes in a large unit in England: A time series analysis
Source: PLoS One. 2021 Mar 31;16(3):e0249233. doi: 10.1371/journal.pone.0249233 (PMC8011758; doi:10.1371/journal.pone.0249233)
Supplement: S1 File — (PDF) [file pone.0249233.s001.pdf]

NIHR | Collaboration for Leadership in Applied Health Research and Care West Midlands

**Name of Maternity Unit**	
**Name of NHS Trust**	

1. **How many births did your Unit have in 2018?**

2. **Can you tell us the average number of hours a week of consultant presence you have on Delivery Suite (DS) currently?**

3. **Have you introduced resident consultant cover on DS for the full 168 hrs a week?** (please circle)

Yes (please go to question 3a)
No (please go to question 4)

3a. If yes, when?
Month ______ Year ______

3b. What do you think the benefits are?

3c. Are you planning to continue? (please circle)
Yes (please go to question 6)
No (please got to question 3d)

3d. If you not planning to continue, why?

NIHR | Collaboration for Leadership in Applied Health Research and Care West Midlands

4. If you have not introduced resident consultant cover for the full 168 hrs a week, can you tell us why?

5. Have you introduced resident consultant cover for any continuous 24 hour period?

Yes (please go to question 5a)
No (please go to question 6)

5a. If yes, how many days on average each week are covered? (please circle) 1 2 3 4 5

5b. Which days are routinely covered? (please circle)

Monday Tuesday Wednesday Thursday Friday Saturday Sunday

5c. What do you think the benefits are?

6. Please tell us any other comments

Thank you for completing the survey
Please return by email to s.kenyon@bham.ac.uk
Or to Professor Sara Kenyon
Freepost RTHB-HUCG-AZYS
Institute of Applied Health Research, Learning Centre, University of Birmingham, B15 2TT
